# Study on Epoxy Resin Toughened by Epoxidized Hydroxy-Terminated Polybutadiene

**DOI:** 10.3390/ma11060932

**Published:** 2018-05-31

**Authors:** Zhen Ge, Wenguo Zhang, Chao Huang, Yunjun Luo

**Affiliations:** School of Materials Science and Engineering, Beijing Institute of Technology, Beijing 100081, China; winger94@163.com (W.Z.); hcatbitmse1146@163.com (C.H.)

**Keywords:** epoxidized hydroxy-terminated polybutadiene, epoxy resin, toughen, mechanical properties

## Abstract

Epoxy resin (EP) was toughened by epoxidized hydroxy-terminated polybutadiene (EHTPB), with the corresponding modified epoxy resin being prepared. In this paper, the microstructure of EHTPB-modified epoxy resin was characterized, while the influence of different contents of EHTPB on curing kinetics, mechanical properties, morphology, thermal properties, dynamic thermomechanical (DMA) properties and crosslink density of the modified epoxy resin were also discussed. The results showed that the EHTPB-modified epoxy resin was successfully prepared and cured completely. The activation energy (*E_a_*) of the modified epoxy resin decreased after the addition of EHTPB. With an increase in the EHTPB content, the tensile strength (σ_m_) of the modified epoxy resin decreased and the breaking elongation (ε_b_) increased gradually. The initial decomposition temperature (T_5%_) and glass transition temperature (T_g_) of the modified epoxy resin decreased with an increase in the EHTPB content. The modified epoxy resin had a rough fractured surface and the interface was blurred, presenting a ductile fracture.

## 1. Introduction

Epoxy resin (EP) is a material with excellent properties, which is widely used in semiconductor equipment manufacturing [1,2,3], adhesives [4,5], aerospace [6,7,8] and other fields. Epoxy resin based on the diglycidylether of bisphenol A (DGEBA) exhibited good mechanical and thermal properties, high chemical and corrosion resistance properties as well as excellent adhesion properties because of the presence of hydroxyl and epoxy groups of EP. However, the brittleness of epoxy resin after curing has limited its application [9]. Therefore, the addition of other materials to strengthen the epoxy resin has been an important research field.

The addition of flexible rubber components to EP (e.g., toughening) is a common method for improving the brittleness of epoxy resin, especially liquid rubbers, such as carboxy-terminated butadiene acrylonitrile (CTBN) rubbers, amino-terminated butadiene acrylonitrile (ATBN) rubbers, hydroxy-terminated butadiene acrylonitrile (HTBN) rubbers, vinyl-terminated butadiene acrylonitrile (VTBN) rubbers, epoxy-terminated butadiene acrylonitrile (ETBN) rubbers and hydroxy-terminated polybutadiene (HTPB). Kargarzadeh [10] studied the toughening mechanism of epoxy resin and proposed two main toughening mechanisms: the mechanism of shear yielding and the mechanism of rubber particle cavitation. Srivastava [11] found that the addition of 15 wt % CTBN could improve the thermal stability of epoxy resin and reduce the curing time, while the generation of the rubber particle cavitation was able to improve the toughness of epoxy resin. Chikhi [12] concluded that as the ATBN content increased, the tensile modulus of the modified epoxy resin decreased and the breaking elongation increased, suggesting that the rubber particle cavitation and the shear yielding was the prevailing toughening mechanisms according to SEM analysis. Wang [13] studied the mechanical influence of HTBN on epoxy resin and found that the impact strength and breaking elongation of epoxy resin with 20 wt % HTBN increased by 28.5% and 46.8%, respectively. Fakhar [14] discovered that when the additional content of VTBN was less than 15 wt %, the fracture toughness and impact resistance of the epoxy resin were improved without a significant reduction in the other mechanical properties or thermal properties. Robinette [15] observed a nine-fold increase in fracture toughness with limited detraction of mechanical properties for the 8 wt % ETBN-modified EP system. Zhou [16] found that the modified epoxy resin with 10 wt % HTPB exhibited an optimal balance of mechanical properties (tensile strength of 67.31 MPa and breaking elongation of 7.75%), but a further increase in HTPB content would result larger rubber particles, thereby weakening the mechanical properties of modified epoxy resin. Otherwise, the addition of carbon nanotubes (CNTs) is another method for toughening the epoxy resin. Barra [17] prepared a series of polyhedral oligomeric silsesquioxanes compounds with the addition of multi-wall carbon nanotubes (MWCNTs) to epoxy formulation and found that the epoxy adhesive containing MWCNTs and glycidyl oligomeric silsesquioxanes presented the best performance in the lap shear strength (LSS) test. Vertuccio [18] designed and characterized new electrical conductive adhesives based on MWCNTs and functionalized liquid rubber. MWCNTs embedded in the epoxy adhesive were able to further increase the LSS of the joints, reinforcing the strong effect caused by rubber domains dispersed in the formulation.

Epoxidized hydroxy-terminated polybutadiene (EHTPB) is a novel polymer obtained by partial double bond epoxidation of the hydroxy-terminated polybutadiene (HTPB) [19,20]. Since the EHTPB retained the flexible segment of HTPB, the polyurethane material based on EHTPB had good toughness, adhesive strength and anti-aging properties. At the same time, the epoxy groups would increase miscibility with the epoxy resin according to the similar compatibility principle. Therefore, if EHTPB was introduced into the epoxy resin matrix, the toughness of epoxy resin might be improved. However, to our knowledge, the investigation of EHTPB toughening epoxy resin was not reported.

In this paper, a polyurethane prepolymer was firstly synthesized by epoxidized hydroxy-terminated polybutadiene (EHTPB) and toluene diisocyanate (TDI), before the prepolymer was introduced into the epoxy resin. The microstructure of EHTPB-modified epoxy resin was characterized by FTIR. The effects of different contents of EHTPB on the curing kinetics, mechanical properties, morphology, thermal properties, dynamic thermomechanical properties and crosslink density of EHTPB-modified epoxy resin were discussed. 

## 2. Experimental

### 2.1. Materials

Epoxy resin E-51 (EP) (industrial grade, molecular weight of 392.2, epoxy value of 5.10 mmol/g, Blue Star Chemical Co., Ltd., Wuxi, China), epoxidized hydroxy-terminated polybutadiene (EHTPB) (molecular weight of 3280, hydroxyl value of 1.06 mmol/g, epoxy value of 3.26 mmol/g, Liming Chemical Research Institute, Luoyang, China), toluene diisocyanate (TDI) (AR, Sinopharm Chemical Reagent Beijing Co., Ltd., Beijing, China), polyamine (A60) (industrial grade, amine value of 460 mg KOH/g, Changsha Chemical Industry Research Institute, Hunan, China), ethyl acetate (AR, Beijing Tongguang Fine Chemicals Company, Beijing, China), diisooctyl sebacate (DOS) (AR, Tianjin Guangfu Fine Chemical Research Institute, Tianjin, China) were dried by standard methods [21]. Dibutyltin dilaurate (AR, Sinopharm Chemical Reagent Beijing Co., Ltd., Beijing, China) was dissolved in DOS to prepare 5 wt ‰ solution (T-12). 

### 2.2. Preparation of EHTPB Modified Epoxy Resin

#### 2.2.1. Preparation Principle

**Figure 1:**
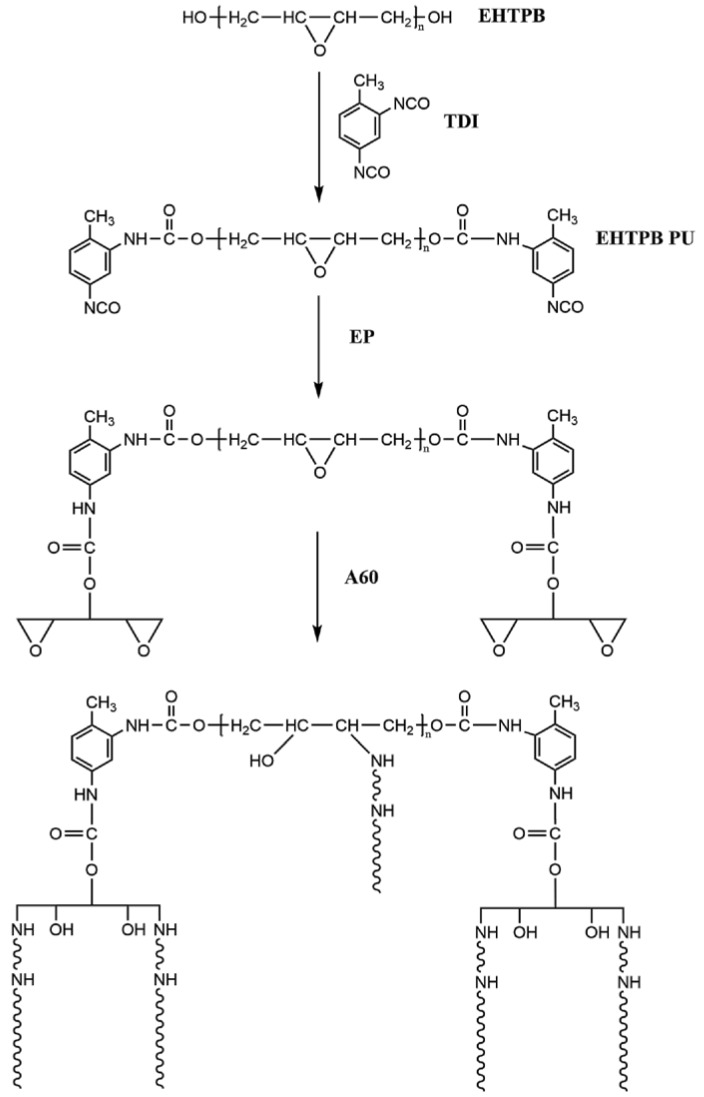
Preparation principle of the EHTPB-modified epoxy resin.

The preparation principle is shown in Figure 1.

#### 2.2.2. Preparation Procedure

**Scheme 1:**
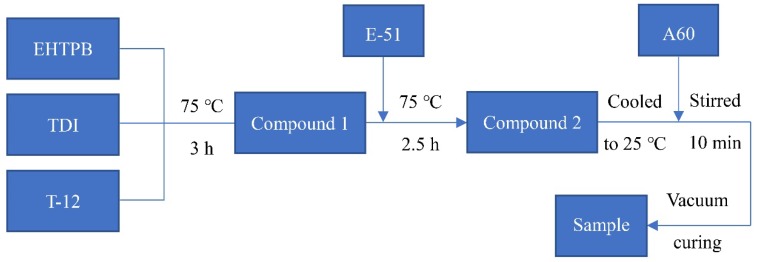
Preparation procedure of the EHTPB-modified epoxy resin.

T-12, which was 1 wt % of the total mass of EHTPB and TDI, was added to a three-neck flask containing ethyl acetate. After mixing, a certain amount of EHTPB was added. After this was dissolved completely, TDI was added dropwise. The pre-polymerization of compound **1** (–NCO (from TDI)/–OH (from EHTPB) mole ratio is 2) was carried out at 75 °C for 3 h. After this, the ethyl acetate solution of epoxy resin E-51 was added to the flask and the reaction was continued for 2.5 h to obtain compound **2**. The mixture was cooled to room temperature. A constant amount of curing agent A60 was added to the cooled prepolymer solution, which was stirred for 10 min. The solution was transferred to a polytetrafluoroethylene mold and the solvent was removed at room temperature under a vacuum, with the sample film obtained after vacuum curing. The EHTPB-modified epoxy resin containing 5–20 wt % EHTPB was prepared according to the procedure shown in Scheme 1. For the neat epoxy resin (containing 0 wt % EHTPB) system, a certain amount of EP was added to a three-neck flask containing ethyl acetate. After this was dissolved completely, a constant amount of A60 was added to the EP solution, which was stirred for 10 min. The subsequent curing procedure was the same as the modified epoxy resin.

### 2.3. Characterization and Analysis

#### 2.3.1. FTIR Analysis

Fourier-transform infrared (FTIR) spectra of the prepared samples were recorded on an infrared spectrophotometer (Thermo Electron Corporation, Nicolet 8700, Shanghai, China) in the wavelength range of 500–4000 cm^−1^. Potassium bromide (KBr) and the samples were grinded, before being pressed in the IR pellet die at 40 psi pressure, before the pellet was placed near the window of FTIR spectrophotometer. The changes in the structure of the polymer at the molecular level were studied from the recorded spectra using a computerized recorder. 

#### 2.3.2. Curing Kinetics Analysis

The curing behavior of EHTPB/EP system and the corresponding curing kinetics parameters were studied by dynamic differential scanning calorimetry (DSC) (METTLER TOLEDO, DSC STARe System, Zurich, Switzerland). The samples were subjected to scanning rates of 5 K/min, 10 K/min, 15 K/min and 20 K/min in the test temperature range of 50–300 °C with a nitrogen flow rate of 40 mL/min. 

The curing kinetics parameters of the system were studied by the Kissinger method, the Ozawa method and the Crane method [22,23,24], such as activation energy (*E_a_*) and reaction order (*n*). The activation energy was an important parameter to determine whether the curing reaction could be carried out. The curing mechanism could be deduced by the reaction order.

*E_a_* could generally be obtained by the Kissinger Equation (1):

(1) $$\ln\left(\beta /T_{p}^{2}\right)=\ln\left(\frac{AR}{E_{a}}\right)-\frac{E_{a}}{R}\left(\frac{1}{T_{p}}\right),$$

where *β* is the heating rate, *T_p_* is the temperature, *R* is the ideal gas constant of 8.314 J/(mol·K), *E_a_* (J/mol) is the activation energy and *A* is the pre-exponential factor. *E_a_* could be also obtained by the Ozawa Equation by Gaussian fitting of ln(*β*/*T_p_*^2^) − 1/*T_p_*.

Reaction order *n* could be obtained by the Crane Equation (2):

(2) $$\frac{d\left(\ln\beta \right)}{d\left(1/T_{p}\right)}=-\left(\frac{E_{a}}{nR}+2T_{p}\right).$$

In the Crane Equation, 2*T_p_* could be neglected since *E_a_/nR* was much larger than 2*T_p_* and thus, the Crane Equation was simplified to Equation (3):

(3) $$\frac{d\left(\ln\beta \right)}{d\left(1/T_{p}\right)}=-\frac{E_{a}}{nR}.$$

Reaction order could be obtained by Gaussian fitting of ln *β* −1/*T_p_* and *E_a_* (the average number of *E_a_* obtained by the Kissinger Equation and the Ozawa Equation) into the Crane Equation.

#### 2.3.3. Mechanical Properties 

The mechanical properties were conducted on the AGS-J Electronic Universal Testing Machine (Shimadzu Corporation, Kyoto, Japan) according to the Chinese National Standard GB/T 528-1998. 

#### 2.3.4. SEM Measurement

The micrographs were obtained on a scanning electron microscopy (SEM) (Hitachi, TM3000, Tokyo, Japan). The fracture surface of the stretched EP samples after tensile testing and the fracture surface of the unstretched EP samples under liquid nitrogen conditions before tensile testing were observed respectively to determine the morphology of the fracture surface.

#### 2.3.5. Thermal Property

The thermal property of the modified epoxy resin was investigated by thermal gravimetric analyzer (TGA) (METTLER TOLEDO, TGA STARe System, Zurich, Switzerland) at the heating rate of 10 K/min from 30 °C to 600 °C with a nitrogen flow rate of 40 mL/min.

#### 2.3.6. DMA

Dynamic thermomechanical analysis (DMA) (METTLER TOLEDO, DMA STARe System, Zurich, Switzerland) of the modified epoxy resin was carried out in shearing mode with the frequency of 10 Hz, the temperature ramp of 3 K/min and scanning range from −50 °C to 200 °C.

#### 2.3.7. Crosslink Density 

The samples of the modified epoxy resin were cut into 10 mm × 5 mm × 2 mm uniform specimens. These specimens were weighed and immersed in pyridine at room temperature for about 5–7 days. After this, the specimens were removed from the solvent, blotted and weighed in a stoppered weighing bottle. 

The crosslink density of the modified epoxy resin was obtained using the equilibrium volume swelling method, according to the Flory–Rehner Equation (4) [25,26]:

(4) $$\nu_{e}=-\frac{\left[\ln\left(1-\nu_{2}\right)+\nu_{2}+\chi \nu_{2}^{2}\right]}{\nu \left(\nu_{2}^{1/3}-2\nu_{2}/f\right)}=\frac{\rho_{b}}{M_{c}},$$

where *ν_e_* was the crosslink density, *ν*_2_ was the volume fraction of the elastomer phase in the swollen elastomer, *ν* was the molar volume of the solvent, *χ* was the interaction parameter between the elastomer and the solvent, *f* was the functionality of the elastomeric network, *ρ_b_* was the density of the elastomer and *M_c_* was the relative average molecular weight of the elastomer.

The *ν*_2_ from Equation (4) can be calculated according to Equation (5):

(5) $$\nu_{2}=\frac{V_{p}}{V_{p}+V_{s}},$$

where *V_p_* was the polymer volume and *V_s_* was the volume of solvent in the swollen elastomer.

The interaction parameter *χ* between the elastomer and the solvent was determined by Equation (6):

(6) $$\chi =0.34+\frac{\nu }{RT}{\left(\delta_{p}-\delta_{s}\right)}^{2},$$

where *δ_p_* and *δ_s_* were the solubility parameters of the elastomer and the solvent, respectively, *R* was the ideal gas constant, *T* was the absolute temperature and *ν* was the volume of the solvent in the swollen elastomer. The solubility parameter *δ_p_* of the elastomer could be determined by the swelling method [27].

## 3. Results and Discussion

### 3.1. FTIR Analysis

**Figure 2:**
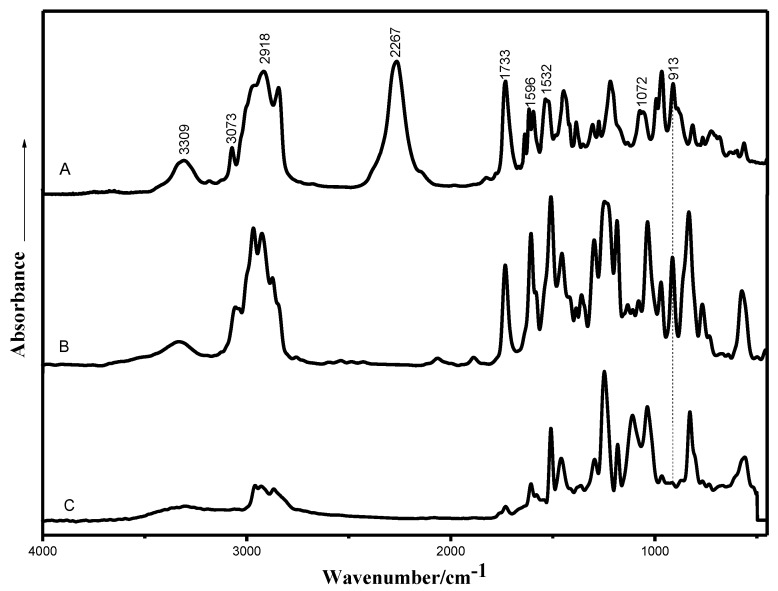
FTIR spectra of the prepared samples (A: EHTPB-based prepolymer; B: EHTPB/EP system before curing; and EHTPB/EP system after curing).

Figure 2 shows the FTIR spectra of the EHTPB-based prepolymer and the EHTPB-modified epoxy resin. For the spectrum of the EHTPB-based prepolymer (curve A), the band located at 3309 cm^−1^ was due to the stretch vibration of N–H, while the band located at 3073 cm^−1^ and 2918 cm^−1^ was attributed to the stretch vibration of the unsaturated –CH and saturated –CH, respectively. The characteristic absorption band of –NCO appeared around 2267 cm^−1^. The band located at 1733 cm^−1^ represented the stretch vibration of C=O, the band located at 1596 cm^−1^ was due to the benzene ring and the absorption at 1072 cm^−1^ was attributed to the stretch vibration of C–O–C. The carbamate characteristic absorption bands located at 3309 cm^−1^, 1733 cm^−1^ and 1532 cm^−1^ indicated that the hydroxy groups reacted with the isocyanate groups to form the polyurethane prepolymer containing carbamate groups [28].

For the spectrum of the EHTPB/EP system before curing (curve B), the –NCO characteristic absorption at 2267 cm^−1^ disappeared, indicating that the isocyanate groups in the polyurethane prepolymer reacted with the secondary hydroxy groups on the epoxy resin to form an epoxy resin–polyurethane graft copolymer.

For the spectrum of the EHTPB/EP system after curing (curve C), the characteristic absorption band at 913 cm^−1^ attributed to the epoxy groups of the EHTPB and the EP [19] disappeared, indicating that the epoxy groups reacted with the amine-based curing agent and the resin system was cured. 

### 3.2. Curing Kinetics Analysis

**Figure 3:**
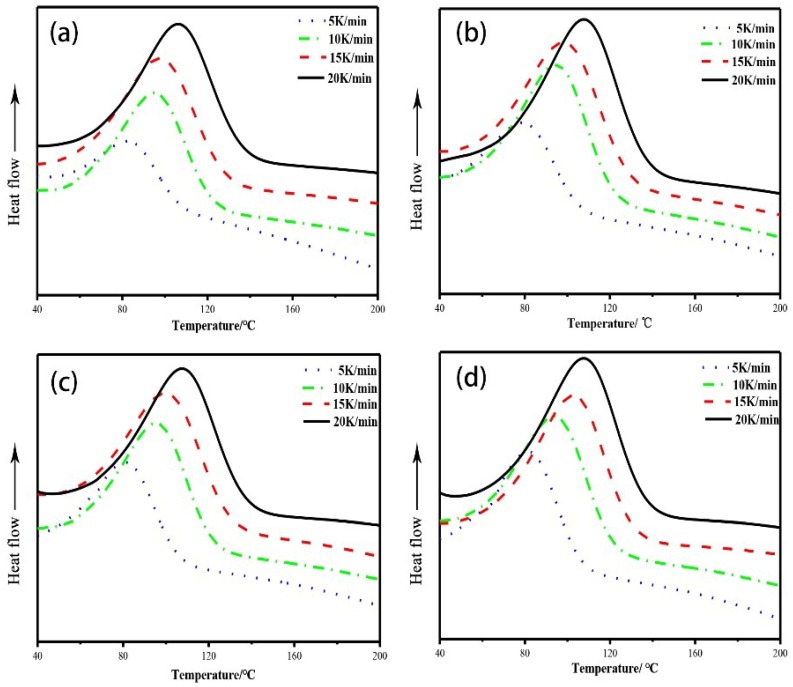

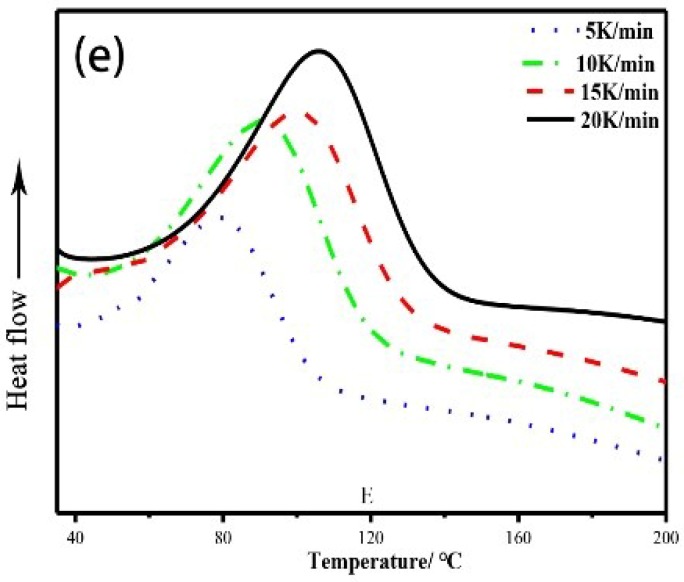
DSC curves of the curing reaction of the modified epoxy resin with different w(EHTPB)/w(EP) ratios: (**a**) w(EHTPB):w(EP) = 0:100; (**b**) w(EHTPB):w(EP) = 5:95; (**c**) w(EHTPB):w(EP) = 10:90; (**d**) w(EHTPB):w(EP) = 15:85; and (**e**) w(EHTPB):w(EP) = 20:80.

Figure 3 shows the DSC curves of the modified epoxy resin with different w(EHTPB)/w(EP) ratios at different heating rates. The curing kinetics parameters are given in Table 1. The *E_a_* values obtained by the Ozawa Equation were similar to those calculated by the Kissinger Equation.

The activation energy of the curing reaction was an important parameter reflecting the reactivity of the curing system, which determined whether the curing reaction could proceed successfully. It can be seen from Table 1 that the activation energy of the epoxy resin system with EHTPB-based polyurethane prepolymer was decreased compared to the neat epoxy resin system. This could be due to two reasons. Firstly, since the EHTPB-based polyurethane prepolymer and the epoxy resin produced hydroxy groups during the curing process, this could promote the epoxy–amine reaction [29]. At the same time, in the case of using the EHTPB-based polyurethane prepolymer-modified epoxy resin, the catalyst T-12 was added to the system in order to accelerate the reaction rate between the isocyanate groups of the polyurethane prepolymer and the hydroxy groups of the epoxy resin, which could reduce the activation energy of the curing reaction of epoxy resin [30]. The change of the activation energy of the modified epoxy resin with different EHTPB contents was not obvious as the standard deviation (except the neat resin) of *E_a_*(avg) was 1.37. This might be due to the same addition content of the T-12 catalyst, which affected the activation energy primarily. The curing reaction order was determined by the chemical reaction type and the effects of each reaction in the reaction process, which subsequently reflects the change of the curing reaction mechanism. As seen from Table 1, the reaction orders of different systems were close to 1 for the first-order reaction, which indicated that the addition of the EHTPB did not change the curing mechanism of EP. It is well known that the curing reactions of epoxy resins with amine curing agents are very complex because many reactive processes simultaneously occur. Three primary curing mechanisms were proposed: (1) primary amine addition; (2) secondary amine addition; and (3) etherification [31]. The first-order reaction indicated that primary amine addition was the main reaction mechanism and thus, a hypothesis was proposed: since no stirring proceeded in the curing process, the reaction was primarily controlled by microscopic molecular collision. The reaction between secondary amine and epoxide was hampered by the post-gelation stage, which was caused by the overfast primary amine addition [32].

### 3.3. Mechanical Properties Analysis

**Figure 4:**
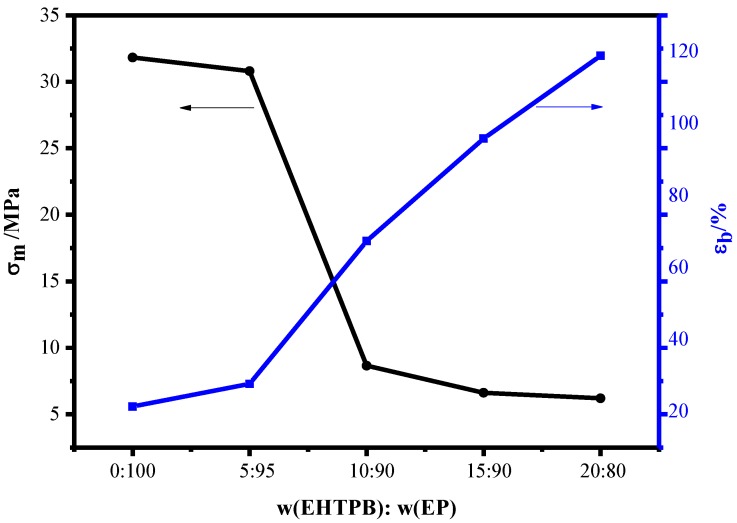
Mechanical properties of the modified epoxy resin with different w(EHTPB)/w(EP) ratios.

The mechanical properties of the modified epoxy resin with different w(EHTPB)/w(EP) ratios are shown in Figure 4. 

Figure 4 shows that with an increase in the EHTPB content, the tensile strength tended to decrease and the breaking elongation increased gradually in the modified epoxy resin. This was due to the formation of the isocyanate-terminated polyurethane prepolymer by the reaction of EHTPB and TDI, with the polyurethane prepolymer able to react with the secondary hydroxy groups of the epoxy resin. Therefore, the rigid epoxy resin had a flexible polyurethane chain. A higher content of EHTPB resulted in a higher content of polyurethane prepolymer in the system, which created more soft segments on the rigid epoxy resin. As a result, the tensile strength decreased and the breaking elongation increased gradually.

**Figure 5:**
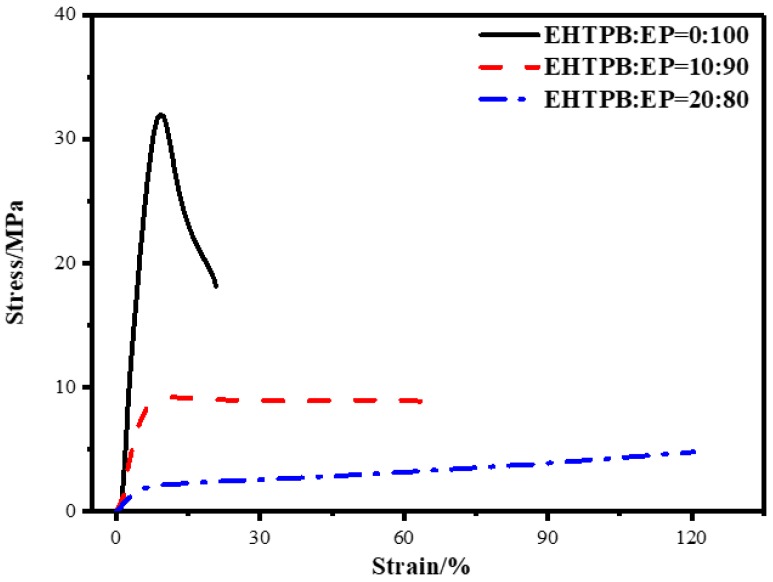
Stress–strain curves of the modified epoxy resin with different w(EHTPB)/w(EP) ratios.

To analyze the overall performance of the modified epoxy resin, the stress–strain curves were obtained by testing data directly, which is shown in Figure 5.

As shown in Figure 5, the curing systems with w(EHTPB)/w(EP) ratios of 0:100, 10:90, 20:80 were selected, and their stress–strain curves were compared. It was found that with an increase in the EHTPB content, the performance of the whole system changed from brittleness (an obvious breaking point of the neat epoxy resin) to ductility. At the same time, when comparing the 20 wt % EHTPB system with the 10 wt % EHTPB system, we found that although there was a tensile strength loss of 28.41% in the 20 wt % EHTPB system, the elongation increased by 76.41%, showing a greater increase in toughness.

### 3.4. SEM Analysis

**Figure 6:**
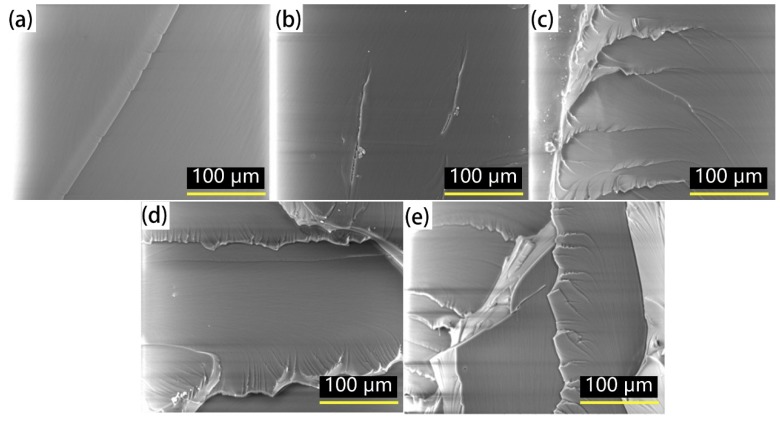
Micrographs of the fracture surface of the stretched modified epoxy resin with different w(EHTPB)/w(EP) ratios after tensile testing: (**a**) w(EHTPB):w(EP) = 0:100; (**b**) w(EHTPB):w(EP) = 5:95; (**c**) w(EHTPB):w(EP) = 10:90; (**d**) w(EHTPB):w(EP) = 15:85; and (**e**) w(EHTPB):w(EP) = 20:80.

**Figure 7:**
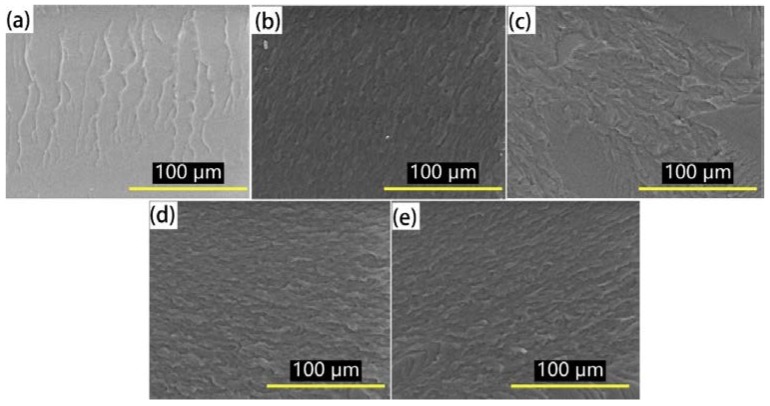
Micrographs of the fracture surface of the unstretched modified epoxy resin with the different w(EHTPB)/w(EP) ratios under liquid nitrogen conditions before tensile testing: (**a**) w(EHTPB):w(EP) = 0:100; (**b**) w(EHTPB):w(EP) = 5:95; (**c**) w(EHTPB):w(EP) = 10:90; (**d**) w(EHTPB):w(EP) = 15:85; and (**e**) w(EHTPB):w(EP) = 20:80.

The micrographs of the modified epoxy resin were obtained by SEM, which are shown in Figure 6 and Figure 7. Figure 6 shows the micrographs of the stretched fracture surface of the modified epoxy resin with different w(EHTPB)/w(EP) ratios magnified by 500 times.

As shown in Figure 6, it was found that the neat epoxy resin had a smooth fracture surface and the fracture direction was concentrated compared with the modified epoxy resin, which presented brittle fracture characteristics. With an increase in the EHTPB content of the modified epoxy resin, the fracture surface gradually blurred and the folds increased, which showed obvious ductile fracture characteristics [33,34].

As the polyurethane prepolymer was grafted to the epoxy resin, the whole system introduced more polyurethane flexible segments. Thus, the flexibility of the entire network structure was improved. When subjected to an external tensile load, the flexible segment itself would yield deformation, which hindered proliferation of microcracks, so the fracture surface showed obvious ductile fracture characteristics. At the same time, with an increase in the EHTPB content, the number of entanglements increased and the damage to this network structure required more stress or energy. Thus, the system exhibited toughness enhancement.

Figure 7 shows the micrographs of the fracture surface of the unstretched modified epoxy resin with different w(EHTPB)/w(EP) ratios under liquid nitrogen conditions before tensile testing. Compared with the modified epoxy resin, the neat epoxy resin showed a single phase structure, while the fracture surface of the modified epoxy resin became rougher and a phase separation (PU/EP two-phase island structure) was observed. The microstructures of the modified epoxy resin with different EHTPB content appeared differently. With an increase in the EHTPB content, the number of island structures increased significantly. This was due to the EHTPB-based polyurethane prepolymer gradually being dispersed out to form the dispersed phase during the curing process, while the epoxy resin formed a continuous phase. From the above analysis, the EHTPB-based polyurethane prepolymer had an outstanding toughness compared to the epoxy resin.

### 3.5. Thermal Analysis

**Figure 8:**
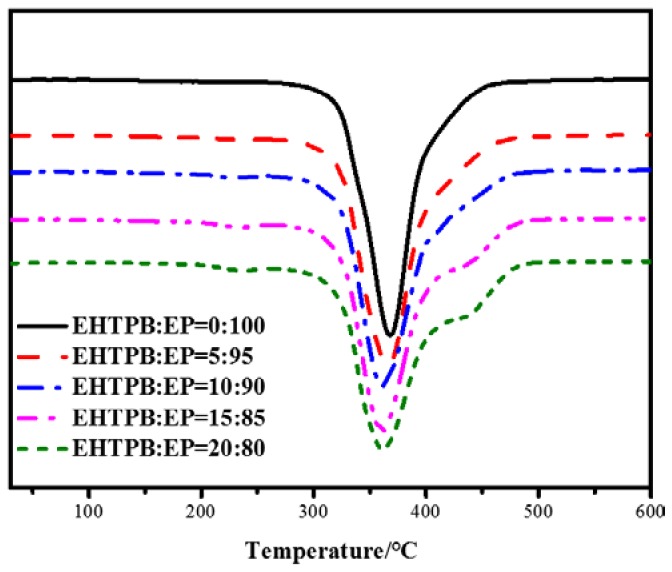
TG curves of the modified epoxy resin with different w(EHTPB)/w(EP) ratios.

**Figure 9:**
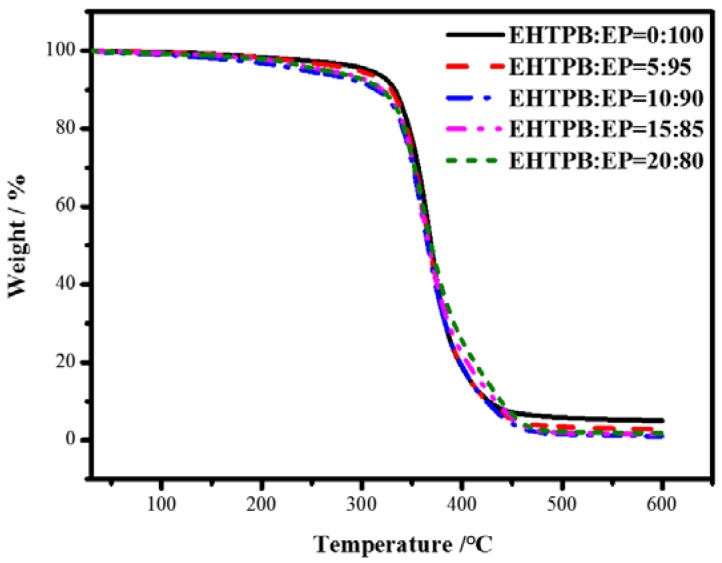
DTG curves of the modified epoxy resin with different w(EHTPB)/w(EP) ratios.

One of the main advantages of epoxy resin is its good thermal properties. The general method of characterizing the thermal property of the material is to use thermal gravimetric analysis (TGA), where the thermal decomposition temperature is the most important parameter. The TG and DTG curves of the modified epoxy resin are shown in Figure 8 and Figure 9. It could be seen from Figure 8 and Figure 9 that the addition of EHTPB-based polyurethane prepolymer did not cause a large change in the curves, indicating that the thermal decomposition mechanism of the modified epoxy resin was almost the same as that of the neat epoxy resin.

The TG and DTG curves of the modified epoxy resin with different w(EHTPB)/w(EP) ratios were analyzed and the corresponding thermal decomposition temperatures were obtained, which are shown in Table 2.

The TG and DTG curves of the neat epoxy resin showed one decomposition stage. The initial decomposition temperature was 307 °C and the temperature at the maximum weight loss rate was 369 °C (Table 2). After adding the EHTPB-based polyurethane prepolymer there were three stages of weight loss according to increasing EHTPB content: the first stage of T_max_ was at about 240 °C, which was due to the decomposition of the carbamate [35]; the second stage of T_max_ was approximately at 360–370 °C, which was due to the chemical bond breaking of epoxy resin backbone [36]; and the third stage of T_max_ was at about 440 °C, which was due to the EHTPB polyurethane backbone chemical bond breaking [37]. The T_5%_ of the modified epoxy resin was lower than that of the neat epoxy resin. The initial decomposition temperature was gradually reduced with an increase in the EHTPB content, which might be attributed to the thermal stability of the polyurethane being lower than that of the epoxy resin.

### 3.6. DMA Analysis

Dynamic thermomechanical analysis (DMA) is a common method for determining the glass transition temperature T_g_, especially when differential scanning calorimetry (DSC) testing is not appropriate due to the glass transition temperature being hard to determine [38].

**Figure 10:**
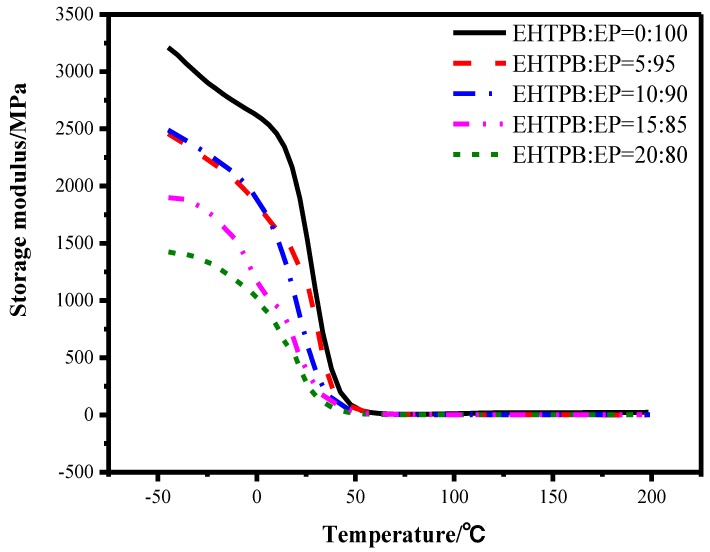
Storage modulus curves of the epoxy resin with different w(EHTPB)/w(EP) ratios.

**Figure 11:**
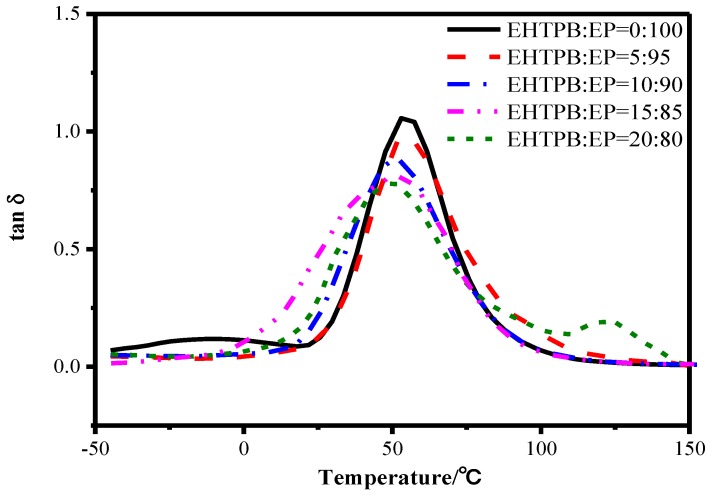
Loss tangent curves of the epoxy resin with different w(EHTPB)/w(EP) ratios.

Storage modulus (E') curves are shown in Figure 10, while loss tangent (tan δ) curves were shown in Figure 11. The data, such as the glass transition temperature, are shown in Table 3.

From Figure 10 and Figure 11 and Table 3, it could be seen that the EHTPB content had a great influence on the storage modulus and glass transition temperature of the EHTPB-modified epoxy resin. The value of T_g_ and tan δ _max_ decreased with an increase in the EHTPB content. This may possibly be due to the polyurethane prepolymer pendant scaffolds having played an internal plasticizing effect in the modified epoxy resin, resulting in a decrease in T_g_. Furthermore, the decrease in T_g_ could also be caused by a decreasing crosslink density [39,40]. It was worth noting that the shape of tan δ peak of the EHTPB/EP (20:80) was not a single peak. Compared with other samples of different EHTPB content, a new peak appeared in 120.2 °C. This phenomenon could be explained as follows: part of the EHTPB prepolymer had been cured separately from the EHTPB/EP curing process to form a curing by-product with high T_g_. We proposed a concept of the ratio of epoxy reaction sites, which was the product of molecular weight and epoxy value. The EHTPB had a higher epoxy reaction sites ratio (3.48 mol/mol) compared to E51 (2.00 mol/mol) by calculation. These reaction sites made the EHTPB curing by-product molecular chain segments rigid and confined the movement of chains. Therefore, a T_g_ peak of the EHTPB curing by-product appeared at 120.2 °C.

The value of tan δ reflected the relationship between the stress and deformation of the material. When the deformation fell behind the stress, the material would lag behind and the corresponding energy would be consumed due to the hysteresis [41,42]. With an increase in the EHTPB content, the flexibility of the molecular chain of the system reduced the force, which hindered the movement of the molecule, and the corresponding tan δ _max_ decreased.

### 3.7. Crosslink Density Analysis

The modified epoxy resin with different EHTPB/EP ratios were tested in pyridine, while the crosslink molecular weight M_c_ and the crosslink density of the modified epoxy resin were calculated by the Flory–Rehner Equation. The final results are shown in Table 4.

It could be seen from Table 4 that the crosslink molecular weight increased gradually from 4022 g/mol to 5686 g/mol, while the crosslink density of epoxy resin E-51 was reduced from 2.83 × 10^−4^ to 2.12 × 10^−4^ mol/g. This was due to the epoxy value of epoxy resin E-51 (5.10 mmol/g) being greater than that of the EHTPB (3.26 mmol/g). Therefore, compared to the same quality of several modified epoxy resin, the total epoxy groups amount of the modified epoxy resin decreased with an increase in the EHTPB content. Thus, the modified epoxy resin crosslink network reduced, reducing the crosslink density, while the molecular weight became larger, allowing more solvent to enter the modified epoxy resin network and causing it to swell until equilibrium.

## 4. Conclusions

The modified epoxy resin with different EHTPB content was successfully prepared. During the curing process, the activation energy of the curing process decreased due to the self-catalysis effect and the addition of T-12, while the curing reaction mechanism did not change. With an increase in the EHTPB content, the tensile strength of the modified epoxy resin decreased from 31.83 MPa to 6.20 MPa and the breaking elongation increased from 21.43% to 118.76%. Compared with the neat epoxy resin, the fracture surface of the modified epoxy resin toughened with EHTPB was rough and the interface was blurred, presenting a ductile fracture. For the fracture behaviors under liquid nitrogen conditions, the modified epoxy resin toughened with EHTPB presented a phase separation, showing island structures. Furthermore, the thermal stability and the glass transition temperature were reduced. The equilibrium volume swelling method showed that the crosslink density decreased from 2.83 × 10^−4^ mol/cm^3^ to 2.12 × 10^−4^ mol/cm^3^.

In short, the addition of an appropriate amount of EHTPB could significantly enhance the toughness of epoxy resin. The preparation of the modified epoxy resin had the characteristics of low curing time and low curing temperature. Therefore, this is an ideal surface coating matrix material, which would attract the attention of researchers worldwide. 

## Figures and Tables

**Table 1:** Curing kinetic parameters of modified epoxy resin with different w(EHTPB)/w(EP) ratios.

w(EHTPB):w(EP)	*E_a_* (kJ/mol)			*n*
	*E_a_* (Kissinger)	*E_a_* (Ozawa)	*E_a_* (avg)	
0:100	63.43	66.40	64.92	0.93
5:95	49.83	53.29	51.56	0.92
10:90	52.30	55.37	53.84	0.92
15:85	52.87	56.14	54.51	0.92
20:80	52.47	55.88	54.18	0.92

**Table 2:** Decomposition temperatures of the modified epoxy resin with different w(EHTPB)/w(EP) ratios.

w(EHTPB):w(EP)	T_5%_ (°C)	T_max_ (°C)		
		Stage 1	Stage 2	Stage 3
0:100	307	-	369	-
5:95	295.5	-	364.3	-
10:90	271.7	243.7	360.1	-
15:85	263.3	242.5	359.6	440.2
20:80	262.2	240.1	361.6	440.7

T_5%_: the initial decomposition temperature; T_max_: the decomposition temperature at the maximum weight loss rate.

**Table 3:** DMA data of the epoxy resin with different w(EHTPB)/w(EP) ratios.

w(EHTPB):w(EP)	tan δ _max_	T_g_ (°C)
0:100	1.06	53.0
5:95	0.98	52.2
10:90	0.89	51.1
15:85	0.83	49.8
20:80	0.78	47.2

**Table 4:** M_c_, crosslink density and density of the modified epoxy resin with different w(EHTPB)/w(EP) ratios.

w(EHTPB):w(EP)	0:100	5:95	10:90	15:85	20:80
M_c_ (g/mol)	4022	4120	4300	4973	5686
ν_e_ × 10^−4^ (mol/cm^3^)	2.83	2.77	2.65	2.39	2.12
Density (g/cm^3^)	1.1389	1.1411	1.1401	1.1875	1.2075
